# Crystal structure of 4-amino-5-fluoro-2-oxo-2,3-di­hydro­pyrimidin-1-ium 3-hy­droxy­pyridine-2-carboxyl­ate

**DOI:** 10.1107/S1600536814021898

**Published:** 2014-10-11

**Authors:** Ammasai Karthikeyan, Packianathan Thomas Muthiah, Franc Perdih

**Affiliations:** a School of Chemistry, Bharathidasan University, Tiruchirappalli 620 024, Tamilnadu, India; b Faculty of Chemistry and Chemical Technology, University of Ljubljana, Vecna pot 113, PO Box 537, SI-1000 Ljubljana, Slovenia; c CO EN-FIST, Trg Osvobodilne fronte 13, SI-1000 Ljubljana, Slovenia

**Keywords:** crystal structure, anti­fungal drug, 5-fluorocytosine, hydrogen bonding, supra­molecular structure, hydrogen-bond ring motifs, crystal structure

## Abstract

The asymmetric unit of the title salt, C_4_H_5_FN_3_O^+^·C_6_H_4_NO_3_^−^, contains one 4-amino-5-fluoro-2-oxo-2,3-di­hydro­pyrimidin-1-ium (5-fluoro­cytosinium, 5FC) cation and a 3-hy­droxy­picolinate (3HAP) anion. The 4-amino-5-fluoro-2-oxo-2,3-di­hydro­pyrimidine mol­ecule is protonated at one of the pyrimidine N atoms. The typical intra­molecular N—H⋯F and O—H⋯O *S*(5) and *S*(6) hydrogen-bond ring motifs are observed in the cations and anions. The protonated N atom and 2-amine group of the 5FC cation inter­act with the 3HPA anion through a pair of nearly parallel N—H⋯O hydrogen bonds, forming a robust *R*_2_^2^(8) ring motif. The ions are further linked by N—H⋯N, O—H⋯O, N—H⋯O and C—H⋯O hydrogen bonds, generating *R*_2_^2^(7), *R*_3_^3^(12) and *R*_6_^5^(18) ring motifs, respectively, leading to supra­molecular wave-like sheets parallel to (010). The crystal structure is further stabilized by C—H⋯π inter­actions, generating a three-dimensional architecture.

## Abstract 2

The protonated N atom and 2-amine group of the 5-fluoro­cytosinium (5FC) cation inter­act with the 3-hy­droxy­picolinate (3HAP) anion through a pair of nearly-parallel N—H⋯O hydrogen bonds, forming a robust 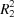
(8) ring motif. The ions are further linked by N—H⋯N, O—H⋯O, N—H⋯O and C—H⋯O hydrogen bonds, leading to supra­molecular wave-like sheets and the crystal structure is further stabilized by C—H⋯π inter­actions, generating a three-dimensional architecture.

## Chemical context   

Fluorinated pyrimidine and purine derivatives have received much inter­est because of their wide range of biological applications (Giner-Sorolla & Bendich, 1958 ▶). 5-Fuoro­cyto­sine is a fluorinated pyrimidine derivative anti-metabolite drug and is also extensively used as an anti-fungal agent for the treatment of *Candida* and *Cryptococcus* (Vermes *et al.*, 2000 ▶). 5-Fluorocytosine is a versatile mol­ecule that plays essential roles in many biological applications, such as anti-tumour, potential gene therapy and gene-directed prodrug therapy (GDEPT) in the treatment of cancer (Kohila *et al.*, 2012 ▶). The crystal structures of 5-fluoro­cytosine monohydrate, 5-fluorocytosine co-crystals and salts have also been reported (Louis *et al.*, 1982 ▶; Tutughamiarso *et al.*, 2012 ▶; Perumalla & Sun, 2014 ▶; Prabakaran *et al.*, 2001 ▶). The crystal structures of various salts and complexes of 3-hy­droxy­picolinic acid have also been reported (Quintal *et al.*, 2000 ▶; Soares-Santos *et al.*, 2003 ▶; Betz and Gerber, 2011 ▶; Nirmalram *et al.*, 2011 ▶). 
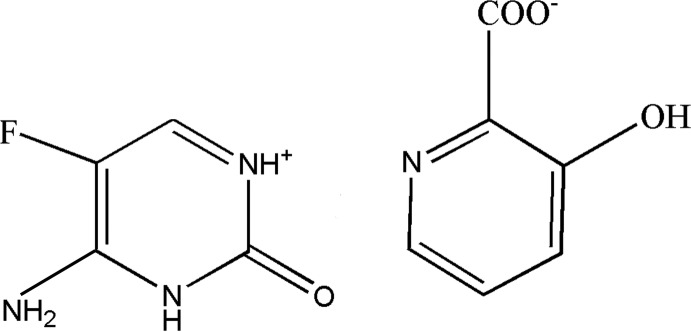


We report herein the mol­ecular structure of the title salt[Chem scheme1], formed from the reaction of 5-fluoro­cytosine with 3-hy­droxy­picoinic acid, namely 5-fluoro­cytosinium 3-hy­droxy­picolinate.

## Structural commentary   

**Figure 1:**
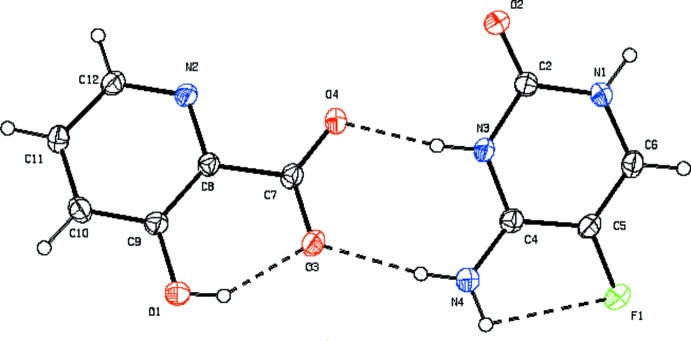
The asymmetric unit of the title compound, showing 30% probability displacement ellipsoids. Dashed lines represent hydrogen bonds.

The asymmetric unit contains a 5-fluoro­cytosinium cation and a 3-hy­droxy­picolinate anion (Fig. 1 ▶). The 5-fluoro­cytosine mol­ecule is protonated at N3, as is evident from the increase in the inter­nal angle at N3 from 120.8 (5) in neutral 5-fluoro­cytosine (Louis *et al.*, 1982 ▶) to 124.85 (15)°. There is an intra­molecular N—H⋯F hydrogen bond with an *S*(5) ring motif between the N4 amino group and the F atom of the 5-fluoro­cytosinum cation. These hydrogen-bonding parameters are similar to those observed in 5-fluoro­cytosinium salicylate (Prabakaran *et al.*, 2001 ▶). An intra­molecular O—H⋯O inter­action forms an *S*(6) motif between the phenolic OH and carboxyl­ate group, which is also observed in 3-hy­droxy­pyridinium-2-carboxyl­ate (Betz & Gerber, 2011 ▶).

## Supra­molecular features   

**Figure 2:**
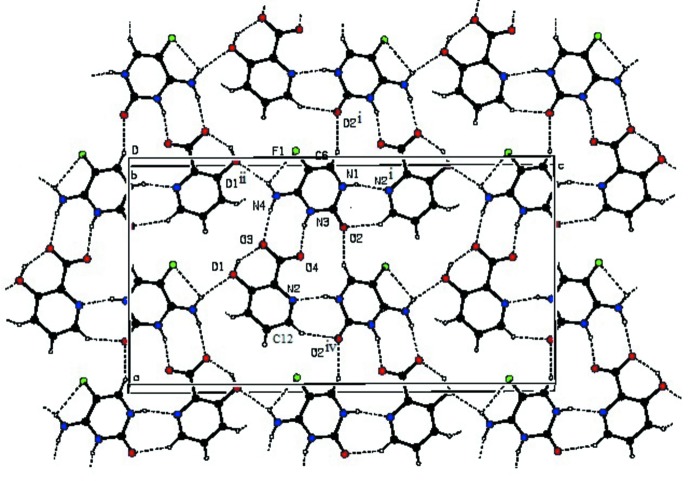
A view of the supra­molecular wavy sheet-like structure formed by N—H⋯F, O—H⋯O, N—H⋯O, N—H⋯N and C—H⋯O hydrogen bonds. Symmetry codes are given in Table 1 ▶. Dashed lines represent hydrogen bonds.

**Figure 3:**
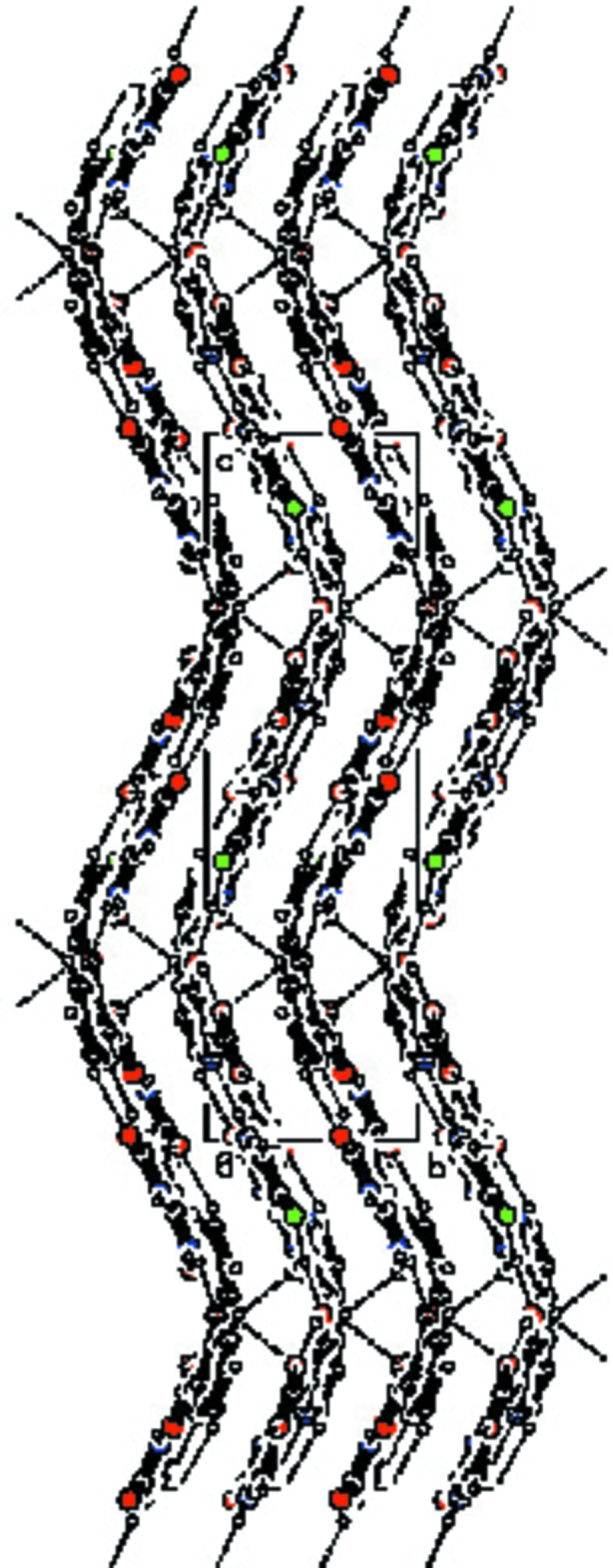
The wavy sheets inter­linked by C—H⋯O hydrogen bonds. Dashed lines represent hydrogen bonds (see Table 1 ▶ for details).

In the crystal structure, the carboxyl­ate group of the 3-hy­droxy­picolinate anion (O3 and O4) inter­acts with the proton­ated N3 atom and the 4-amino group of the 5-fluoro­cytosinium moiety through a pair of N—H⋯O hydrogen bonds, forming a robust 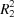
(8) motif (Etter, 1990 ▶; Bernstein *et al.*, 1995 ▶). The 3-hy­droxy­picolinate (N2 and C12) atoms inter­act with the N1 atom and the exocyclic oxygen O2 atom of the 5-fluoro­cytosinium moiety through a pair of N—H⋯N and C—H⋯O hydrogen bonds, forming an 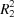
(7) motif. This type of motif rarely occurs in cytosinium carboxyl­ate inter­actions (Benali-Cherif *et al.*, 2009 ▶). The motif is further connected on the other side by 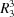
(12) and 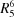
(18) motifs formed (Bernstein *et al.*, 1995 ▶) through C—H⋯O and N—H⋯O hydrogen bonds involving the O2 and N4 atoms of the 5-fluoro­cytosinium cation and the symmetry-related C6 atom of the another cytosinium cation and O1 atoms of 3-hy­droxy­picolinate anions, generating a wavy sheet-like structure parallel to (010) (Fig. 2 ▶). These wavy sheets are inter­connected *via* C10—H10⋯O2 hydrogen bonds (Fig. 3 ▶). The crystal structure is further stabilized by C—H⋯π inter­actions between 3-hy­droxy­picolinate anions, Table 1 ▶.

## Synthesis and crystallization   

Hot aqueous solutions of 5-fluoro­cytosine (32 mg, Alfa Aesar) and 3-hy­droxy­picolinic acid (37 mg, Alfa Aesar) were mixed in a 1:1 molar ratio. The resulting solution was warmed over a water bath for half an hour and then kept at room temperature for crystallization. After a week, colourless prismatic crystals were obtained.

## Refinement   

Crystal data, data collection and structure refinement details are summarized in Table 2 ▶. All H atoms were initially located in difference Fourier maps and were subsequently treated as riding atoms in geometrically idealized positions, with C—H = 0.93, N—H = 0.86 and O—H = 0.83 Å, and with *U*_iso_(H) = 1.2*U*_eq_(C,N) and *U*_iso_(H) = 1.5*U*_eq_(O).

## Supplementary Material

Crystal structure: contains datablock(s) I. DOI: 10.1107/S1600536814021898/tk5344sup1.cif

Structure factors: contains datablock(s) I. DOI: 10.1107/S1600536814021898/tk5344Isup2.hkl

Supporting information file. DOI: 10.1107/S1600536814021898/tk5344Isup3.cml

CCDC reference: 1027535

Additional supporting information:  crystallographic information; 3D view; checkCIF report

## Figures and Tables

**Figure 4:**
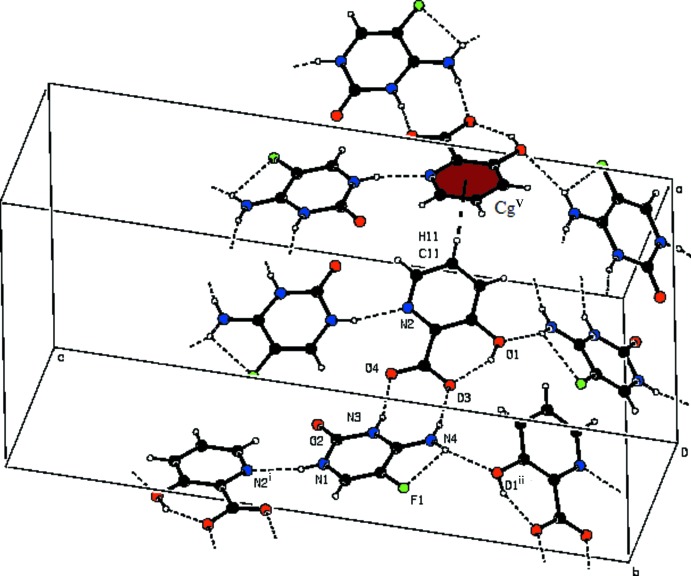
A view of the C—H⋯π inter­actions shown as dashed lines. Symmetry codes are given in Table 1 ▶.

**Table 1:** Hydrogen-bond geometry (, ) *Cg* is the centroid of the N2/C8C12 ring.

*D*H*A*	*D*H	H*A*	*D* *A*	*D*H*A*
N1H1N2^i^	0.86	2.04	2.873(2)	163
N3H3O4	0.86	1.85	2.6665(18)	158
N4H4*A*O3	0.86	1.98	2.830(2)	169
N4H4*B*O1^ii^	0.86	2.26	3.076(2)	159
N4H4*B*F1	0.86	2.43	2.7312(18)	101
O1H1*A*O3	0.82	1.83	2.5542(18)	146
C6H6O2^i^	0.93	2.29	3.127(2)	150
C10H10O3^iii^	0.93	2.54	3.272(2)	136
C12H12O2^iv^	0.93	2.39	3.129(2)	137
CllH11*Cg*1^v^	0.93	2.88	3.426(2)	119

Symmetry codes: (i) 
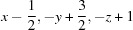
; (ii) 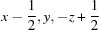; (iii) 
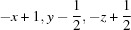
; (iv) 
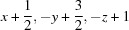
; (v) 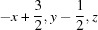.

**Table 2:** Experimental details

Crystal data	
Chemical formula	C_4_H_5_FN_3_O^+^C_6_H_4_NO_3_
*M*_r_	268.21
Crystal system, space group	Orthorhombic, *P* *b* *c* *a*
Temperature (K)	293
*a*, *b*, *c* ()	12.6487(4), 7.0786(2), 23.7200(6)
*V* (^3^)	2123.77(10)
*Z*	8
Radiation type	Mo *K*
(mm^1^)	0.14
Crystal size (mm)	0.15 0.05 0.05

Data collection	
Diffractometer	Agilent SuperNova (Dual, Cu at zero, Atlas)
Absorption correction	Multi-scan (*CrysAlis PRO*; Agilent, 2011 ▶)
*T*_min_, *T*_max_	0.979, 0.993
No. of measured, independent and observed [*I* > 2(*I*)] reflections	8914, 2437, 1955
*R*_int_	0.027

Refinement	
*R*[*F* ^2^ > 2(*F* ^2^)], *wR*(*F* ^2^), *S*	0.042, 0.111, 1.08
No. of reflections	2437
No. of parameters	173
H-atom treatment	H-atom parameters constrained
_max_, _min_ (e ^3^)	0.24, 0.19

Computer programs: *CrysAlis PRO* (Agilent, 2011 ▶), *SHELXS97* and *SHELXL97* (Sheldrick, 2008 ▶) and *PLATON* (Spek, 2009 ▶).

## supplementary crystallographic information

### Crystal data

**Table:**

C_4_H_5_FN_3_O^+^·C_6_H_4_NO_3_^−^	*F*(000) = 1104
*M**_r_* = 268.21	*D*_x_ = 1.678 Mg m^−^^3^
Orthorhombic, *P**b**c**a*	Mo *K*α radiation, λ = 0.71073 Å
Hall symbol: -P 2ac 2ab	Cell parameters from 3551 reflections
*a* = 12.6487 (4) Å	θ = 3.7–29.7°
*b* = 7.0786 (2) Å	µ = 0.14 mm^−^^1^
*c* = 23.7200 (6) Å	*T* = 293 K
*V* = 2123.77 (10) Å^3^	Needle, colourless
*Z* = 8	0.15 × 0.05 × 0.05 mm

### Data collection

**Table:**

Agilent SuperNova (Dual, Cu at zero, Atlas) diffractometer	2437 independent reflections
Radiation source: SuperNova (Mo) X-ray Source	1955 reflections with *I* > 2σ(*I*)
Mirror monochromator	*R*_int_ = 0.027
Detector resolution: 10.4933 pixels mm^-1^	θ_max_ = 27.5°, θ_min_ = 3.2°
ω scans	*h* = −16→11
Absorption correction: multi-scan (*CrysAlis PRO*; Agilent, 2011)	*k* = −9→6
*T*_min_ = 0.979, *T*_max_ = 0.993	*l* = −30→30
8914 measured reflections	

### Refinement

**Table:**

Refinement on *F*^2^	Primary atom site location: structure-invariant direct methods
Least-squares matrix: full	Secondary atom site location: difference Fourier map
*R*[*F*^2^ > 2σ(*F*^2^)] = 0.042	Hydrogen site location: inferred from neighbouring sites
*wR*(*F*^2^) = 0.111	H-atom parameters constrained
*S* = 1.08	*w* = 1/[σ^2^(*F*_o_^2^) + (0.0458*P*)^2^ + 0.8599*P*] where *P* = (*F*_o_^2^ + 2*F*_c_^2^)/3
2437 reflections	(Δ/σ)_max_ < 0.001
173 parameters	Δρ_max_ = 0.24 e Å^−^^3^
0 restraints	Δρ_min_ = −0.19 e Å^−^^3^

### Special details

**Table:**

Experimental. 185 frames in 5 runs of ω scans. Crystal-detector distance = 55.0 mm.
Geometry. All s.u.'s (except the s.u. in the dihedral angle between two l.s. planes) are estimated using the full covariance matrix. The cell s.u.'s are taken into account individually in the estimation of s.u.'s in distances, angles and torsion angles; correlations between s.u.'s in cell parameters are only used when they are defined by crystal symmetry. An approximate (isotropic) treatment of cell s.u.'s is used for estimating s.u.'s involving l.s. planes.
Refinement. Refinement of *F*^2^ against ALL reflections. The weighted *R*-factor *wR* and goodness of fit *S* are based on *F*^2^, conventional *R*-factors *R* are based on *F*, with *F* set to zero for negative *F*^2^. The threshold expression of *F*^2^ > 2σ(*F*^2^) is used only for calculating *R*-factors(gt) *etc*. and is not relevant to the choice of reflections for refinement. *R*-factors based on *F*^2^ are statistically about twice as large as those based on *F*, and *R*- factors based on ALL data will be even larger.

### Fractional atomic coordinates and isotropic or equivalent isotropic displacement parameters (Å^2^)

**Table:**

	*x*	*y*	*z*	*U*_iso_*/*U*_eq_	
F1	−0.04979 (8)	0.58078 (18)	0.39473 (5)	0.0483 (3)	
N1	0.10109 (11)	0.8014 (2)	0.50442 (6)	0.0335 (3)	
H1	0.0909	0.8491	0.5373	0.040*	
N3	0.21136 (11)	0.7249 (2)	0.42943 (6)	0.0312 (3)	
H3	0.2733	0.7239	0.4145	0.037*	
N4	0.14821 (12)	0.5828 (2)	0.34880 (6)	0.0392 (4)	
H4A	0.2108	0.5846	0.3347	0.047*	
H4B	0.0966	0.5360	0.3298	0.047*	
O2	0.27785 (10)	0.8667 (2)	0.50714 (5)	0.0446 (4)	
C2	0.20128 (14)	0.8021 (3)	0.48270 (7)	0.0317 (4)	
C4	0.13147 (14)	0.6513 (2)	0.39922 (7)	0.0296 (4)	
C5	0.03071 (13)	0.6548 (3)	0.42474 (7)	0.0332 (4)	
C6	0.01722 (14)	0.7286 (3)	0.47624 (7)	0.0346 (4)	
H6	−0.0496	0.7299	0.4926	0.041*	
O1	0.49981 (11)	0.4529 (2)	0.24635 (5)	0.0430 (4)	
H1A	0.4410	0.4833	0.2578	0.064*	
O3	0.36400 (10)	0.5738 (2)	0.31741 (5)	0.0408 (3)	
O4	0.41321 (10)	0.6570 (2)	0.40413 (5)	0.0416 (3)	
N2	0.61308 (11)	0.5297 (2)	0.38561 (6)	0.0296 (3)	
C7	0.43231 (13)	0.5915 (3)	0.35634 (7)	0.0301 (4)	
C8	0.54219 (13)	0.5251 (2)	0.34318 (7)	0.0274 (4)	
C9	0.56959 (14)	0.4572 (3)	0.28970 (7)	0.0306 (4)	
C10	0.67182 (14)	0.3927 (3)	0.28059 (7)	0.0347 (4)	
H10	0.6918	0.3464	0.2455	0.042*	
C11	0.74279 (14)	0.3982 (3)	0.32407 (7)	0.0339 (4)	
H11	0.8118	0.3565	0.3189	0.041*	
C12	0.71035 (14)	0.4669 (3)	0.37608 (7)	0.0332 (4)	
H12	0.7589	0.4690	0.4055	0.040*	

### Atomic displacement parameters (Å^2^)

**Table:**

	*U*^11^	*U*^22^	*U*^33^	*U*^12^	*U*^13^	*U*^23^
F1	0.0265 (6)	0.0690 (8)	0.0494 (7)	−0.0079 (5)	−0.0027 (5)	−0.0139 (6)
N1	0.0293 (8)	0.0439 (9)	0.0272 (7)	0.0025 (7)	0.0027 (6)	−0.0036 (6)
N3	0.0221 (7)	0.0427 (8)	0.0288 (7)	−0.0005 (6)	0.0025 (5)	−0.0008 (6)
N4	0.0295 (8)	0.0552 (10)	0.0330 (8)	−0.0036 (8)	0.0024 (6)	−0.0086 (7)
O2	0.0291 (7)	0.0668 (9)	0.0379 (7)	−0.0048 (7)	−0.0022 (5)	−0.0131 (7)
C2	0.0278 (9)	0.0392 (9)	0.0280 (8)	0.0024 (8)	0.0003 (7)	0.0009 (7)
C4	0.0278 (9)	0.0330 (9)	0.0280 (8)	0.0017 (7)	−0.0008 (6)	0.0039 (7)
C5	0.0238 (8)	0.0406 (10)	0.0351 (9)	−0.0010 (8)	−0.0034 (7)	−0.0001 (8)
C6	0.0249 (8)	0.0426 (10)	0.0362 (9)	0.0019 (8)	0.0043 (7)	0.0038 (8)
O1	0.0331 (7)	0.0677 (9)	0.0280 (6)	0.0063 (7)	−0.0043 (5)	−0.0070 (6)
O3	0.0251 (6)	0.0644 (9)	0.0329 (7)	0.0030 (6)	−0.0020 (5)	0.0009 (6)
O4	0.0275 (7)	0.0623 (9)	0.0349 (7)	0.0053 (6)	0.0032 (5)	−0.0091 (6)
N2	0.0244 (7)	0.0380 (8)	0.0265 (7)	−0.0016 (6)	0.0002 (5)	0.0011 (6)
C7	0.0247 (8)	0.0361 (9)	0.0294 (8)	−0.0013 (7)	0.0015 (7)	0.0045 (7)
C8	0.0238 (8)	0.0326 (9)	0.0258 (8)	−0.0020 (7)	0.0014 (6)	0.0024 (7)
C9	0.0292 (9)	0.0367 (9)	0.0258 (8)	−0.0007 (8)	−0.0012 (6)	0.0007 (7)
C10	0.0357 (10)	0.0394 (10)	0.0290 (8)	0.0047 (8)	0.0048 (7)	−0.0026 (7)
C11	0.0257 (9)	0.0375 (9)	0.0386 (9)	0.0058 (8)	0.0028 (7)	0.0011 (8)
C12	0.0267 (9)	0.0411 (10)	0.0319 (9)	0.0005 (8)	−0.0030 (7)	0.0012 (8)

### Geometric parameters (Å, º)

**Table:**

F1—C5	1.348 (2)	O1—C9	1.355 (2)
N1—C6	1.356 (2)	O1—H1A	0.8200
N1—C2	1.368 (2)	O3—C7	1.271 (2)
N1—H1	0.8600	O4—C7	1.248 (2)
N3—C4	1.344 (2)	N2—C12	1.328 (2)
N3—C2	1.383 (2)	N2—C8	1.348 (2)
N3—H3	0.8600	C7—C8	1.500 (2)
N4—C4	1.308 (2)	C8—C9	1.400 (2)
N4—H4A	0.8600	C9—C10	1.388 (2)
N4—H4B	0.8600	C10—C11	1.368 (2)
O2—C2	1.218 (2)	C10—H10	0.9300
C4—C5	1.411 (2)	C11—C12	1.388 (2)
C5—C6	1.340 (2)	C11—H11	0.9300
C6—H6	0.9300	C12—H12	0.9300
			
C6—N1—C2	122.73 (15)	C9—O1—H1A	109.5
C6—N1—H1	118.6	C12—N2—C8	118.75 (14)
C2—N1—H1	118.6	O4—C7—O3	124.40 (16)
C4—N3—C2	124.84 (15)	O4—C7—C8	118.99 (15)
C4—N3—H3	117.6	O3—C7—C8	116.60 (15)
C2—N3—H3	117.6	N2—C8—C9	121.32 (15)
C4—N4—H4A	120.0	N2—C8—C7	116.96 (14)
C4—N4—H4B	120.0	C9—C8—C7	121.70 (15)
H4A—N4—H4B	120.0	O1—C9—C10	118.77 (15)
O2—C2—N1	123.98 (16)	O1—C9—C8	122.25 (16)
O2—C2—N3	120.68 (16)	C10—C9—C8	118.98 (15)
N1—C2—N3	115.34 (15)	C11—C10—C9	118.99 (16)
N4—C4—N3	120.63 (16)	C11—C10—H10	120.5
N4—C4—C5	123.02 (16)	C9—C10—H10	120.5
N3—C4—C5	116.34 (15)	C10—C11—C12	119.08 (16)
C6—C5—F1	122.47 (16)	C10—C11—H11	120.5
C6—C5—C4	120.85 (16)	C12—C11—H11	120.5
F1—C5—C4	116.68 (15)	N2—C12—C11	122.87 (16)
C5—C6—N1	119.89 (16)	N2—C12—H12	118.6
C5—C6—H6	120.1	C11—C12—H12	118.6
N1—C6—H6	120.1		
			
C6—N1—C2—O2	179.95 (18)	C12—N2—C8—C7	178.04 (16)
C6—N1—C2—N3	0.7 (3)	O4—C7—C8—N2	4.0 (2)
C4—N3—C2—O2	−179.68 (17)	O3—C7—C8—N2	−174.95 (16)
C4—N3—C2—N1	−0.4 (3)	O4—C7—C8—C9	−177.49 (17)
C2—N3—C4—N4	179.33 (17)	O3—C7—C8—C9	3.6 (3)
C2—N3—C4—C5	0.0 (3)	N2—C8—C9—O1	−179.39 (16)
N4—C4—C5—C6	−179.16 (18)	C7—C8—C9—O1	2.2 (3)
N3—C4—C5—C6	0.2 (3)	N2—C8—C9—C10	0.3 (3)
N4—C4—C5—F1	0.8 (3)	C7—C8—C9—C10	−178.17 (16)
N3—C4—C5—F1	−179.85 (15)	O1—C9—C10—C11	179.43 (17)
F1—C5—C6—N1	−179.86 (16)	C8—C9—C10—C11	−0.3 (3)
C4—C5—C6—N1	0.1 (3)	C9—C10—C11—C12	0.4 (3)
C2—N1—C6—C5	−0.5 (3)	C8—N2—C12—C11	0.7 (3)
C12—N2—C8—C9	−0.5 (3)	C10—C11—C12—N2	−0.7 (3)

### Hydrogen-bond geometry (Å, º)

Cg is the centroid of the N2/C8–C12 ring.

**Table:**

*D*—H···*A*	*D*—H	H···*A*	*D*···*A*	*D*—H···*A*
N1—H1···N2^i^	0.86	2.04	2.873 (2)	163
N3—H3···O4	0.86	1.85	2.6665 (18)	158
N4—H4*A*···O3	0.86	1.98	2.830 (2)	169
N4—H4*B*···O1^ii^	0.86	2.26	3.076 (2)	159
N4—H4*B*···F1	0.86	2.43	2.7312 (18)	101
O1—H1*A*···O3	0.82	1.83	2.5542 (18)	146
C6—H6···O2^i^	0.93	2.29	3.127 (2)	150
C10—H10···O3^iii^	0.93	2.54	3.272 (2)	136
C12—H12···O2^iv^	0.93	2.39	3.129 (2)	137
Cll—H11···*Cg*1^v^	0.93	2.88	3.426 (2)	119

Symmetry codes: (i) *x*−1/2, −*y*+3/2, −*z*+1; (ii) *x*−1/2, *y*, −*z*+1/2; (iii) −*x*+1, *y*−1/2, −*z*+1/2; (iv) *x*+1/2, −*y*+3/2, −*z*+1; (v) −*x*+3/2, *y*−1/2, *z*.
